# Antibody fragments functionalized with non-canonical amino acids preserving structure and functionality - A door opener for new biological and therapeutic applications

**DOI:** 10.1016/j.heliyon.2023.e22463

**Published:** 2023-11-20

**Authors:** Hana Hanaee-Ahvaz, Monika Cserjan-Puschmann, Florian Mayer, Christopher Tauer, Bernd Albrecht, Paul G. Furtmüller, Birgit Wiltschi, Rainer Hahn, Gerald Striedner

**Affiliations:** a Christian Doppler Laboratory for Production of Next-Level Biopharmaceuticals in *E. coli*, University of Natural Resources and Life Sciences, Vienna, Department of Biotechnology, Institute of Bioprocess Science and Engineering, Muthgasse 18, 1190, Vienna, Austria; b Biopharma Austria, Process Science, Boehringer Ingelheim Regional Center Vienna GmbH & Co KG, Dr.-Boehringer-Gasse 5-11, A-1121, Vienna, Austria; c University of Natural Resources and Life Sciences, Vienna, Department of Chemistry, Institute of Biochemistry, Muthgasse 18, 1190, Vienna, Austria

**Keywords:** *E. coli*, Non-canonical amino acids, Antigen binding fragment, Micro-bioreactor cultivations

## Abstract

Functionalization of proteins by incorporating reactive non-canonical amino acids (ncAAs) has been widely applied for numerous biological and therapeutic applications. The requirement not to lose the intrinsic properties of these proteins is often underestimated and not considered. Main purpose of this study was to answer the question whether functionalization via residue-specific incorporation of the ncAA N^6^-[(2-Azidoethoxy) carbonyl]-l-lysine (Azk) influences the properties of the anti-tumor-necrosis-factor-α-Fab (FTN2). Therefore, FTN2_Azk_ variants with different Azk incorporation sites were designed and amber codon suppression was used for production. The functionalized FTN2_Azk_ variants were efficiently produced in fed-batch like μ-bioreactor cultivations in the periplasm of *E. coli* displaying correct structure and antigen binding affinities comparable to those of wild-type FTN2. Our FTN2_Azk_ variants with reactive handles for diverse conjugates enable tracking of recombinant protein in the production cell, pharmacological studies and translation into new pharmaceutical applications.

## 1 Introduction

The site-specific incorporation of non-canonical amino acids (ncAAs) facilitates specific chemical functionalities to be rationally introduced into proteins. The chemical diversification is currently used for high value proteins and peptides mainly for biopharmaceutical applications (functionalized proteins and peptides, scaffolds for drug candidates) [1,2]. The increased structural and functional diversity of proteins opens up a new dimension of possibilities in chemical and synthetic biology with an abundance of applications in fundamental research and biotechnology such as phage display screening [3,4], identification of antibodies with improved binding properties or specificity [1,5] and identifying the underpinning mechanism of antigen antibody interactions [6].

The site-specific incorporation of ncAAs into proteins requires an orthogonal element consisting of an engineered aminoacyl-tRNA synthetase (aaRS) and its cognate tRNA pair [7,8]. The orthogonal aaRS is designed to selectively recognize and attach the desired ncAA to its corresponding tRNA molecule, forming an aminoacyl-tRNA complex. This orthogonal system operates independently of the cell's natural machinery, ensuring that the ncAA is specifically incorporated at a chosen site within the protein. Typically, the amber stop codon (TAG, the least frequent stop codon) in *E. coli*, is designated for ncAA incorporation. The orthogonal tRNA, carrying the ncAA, recognizes and pairs with this codon during translation, facilitating the site-specific introduction of ncAAs into proteins.

Based on the application, there are two purposes for using ncAAs. First, the physicochemical characteristics of the incorporated ncAA can increase the thermal stability of enzymes [9,10], improve the catalytic activity [11], and improve the binding affinity or specificity of the antibodies. Second, other ncAAs have reactive moieties that are used for expansion of the antibody's chemistry for site-specific payload/stabilizer conjugation. Site-specific conjugations guarantees the homogenous antibody drug conjugate with expected pharmacology regarding the safety profiles and efficacy [12]. Azide is an inert small reactive moiety totally absent in biological systems that can provide the handle for site-specific conjugation [13]. Azide alkyne cycloaddition generates a heterocyclic triazol through strain promoted azide alkyne cycloaddition (spAAc) which is a class of click chemistry. This reaction is mild and can be performed under physiological conditions. In addition to providing a site-specific conjugation possibility, the nature of the linkage between azide and alkyne is extremely stable [14,15]. Therefore, it has been introduced as an efficient tool for potentizing the antibody through payload conjugation or stabilizing the antigen binding fragment (Fab) or other small proteins through polyethylene glycol (PEG) conjugation [15]. Examples of azide containing ncAAs are *N*^6^-((2-azidoethoxy)carbonyl)-l-lysine (Azk), Azidohomoalanine (AHA), *para*-azidomethyl-l-phenylalanine (pAMF) and *para*-azidophenylalanine (pAzF).

In this study, we investigated the impact of Azk incorporation at different sites of the anti-tumor necrosis factor α (TNFα) Fab (FTN2) on its expression, structure, and functionality. Overall, we demonstrated that our concept for identification of incorporation sites resulted in Azk-modified full functional FTN2_Azk_ variants with expression yields close to that of wild type (wt) FTN2. These variants with site-specifically incorporated azides serve as clickable handles for the conjugation of target molecules of interest, including commercially available conjugates such as fluorophores, radioisotopes, PEGs, or drugs enabling tracking of the recombinant protein's fate in the production host cell, supporting pharmacological studies, and facilitating the translation into new pharmaceutical applications.

## 2 Results

The main goal of this study was to design and characterize Fab FTN2 variants functionalized with the ncAA Azk, which are efficiently and correctly expressed in the periplasm of *E. coli* and retain their antigen-binding functionality and structural content. Furthermore, the surface accessibility of the incorporated Azk for subsequent conjugation purposes was investigated. Therefore, different FTN2_Azk_ variants with Azk incorporated at specified sites were generated and evaluated. Each individual FTN2_Azk_ variant was named based on the Azk incorporation site (e.g. E_LC_143Z: FTN2_Azk_ variant with Azk incorporated in the light chain (LC) at position 143). In all experiments, wt FTN2 was included as control.

### 2.1 Design of FTN2_Azk_ variants

**Fig. 1:**
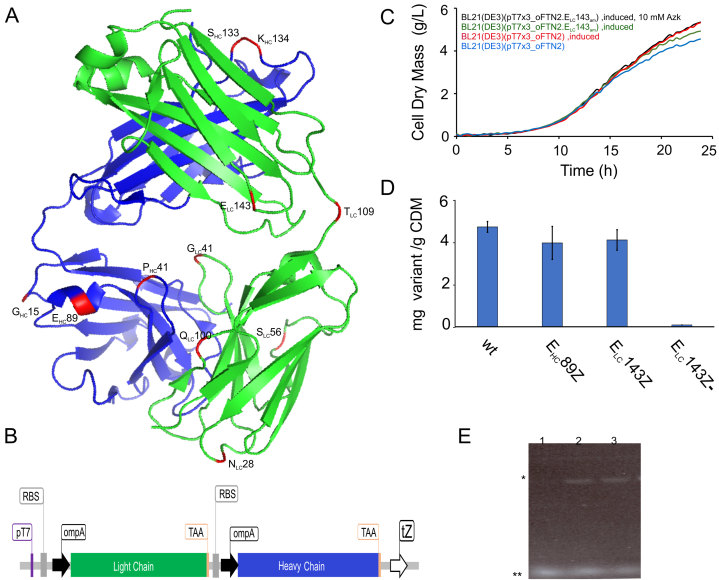
Design and production of Azk-functionalized Fab FTN2 variants. **(A)** Selected positions for Azk incorporation into the Fab FTN2 (PDB:5WUV). The HC is shown in blue and the LC in green; exchanged AAs are shown in red. **(B)** Schematic FTN2 expression cassette and its elements: T7 promotor (pT7), ribosome binding site (RBS), Termination signal T Zenit (tZ) introduced into the (pT7x3) plasmid (shown in Fig. S1). **(C)** Growth kinetics for BL21(DE3) strains carrying different (pT7x3) plasmids in μ-bioreactor cultivations. CDM is calculated from a predefined calibration curve by plotting light scatterd light data against known CDM (**D)** Specific concentration of correctly folded FTN2_Azk_ variants determined by ELISA (n = 2 biological replicates; error bars indicate upper and lower bounds from the mean). (−) indicates that the culture medium was not supplemented with Azk (negative control). See also Fig. S2. **(E)** SDS PAGE of wt FTN2 (lane 1), E_HC_89Z (lane2) and E_LC_143Z (lane3) conjugated with DBCO-AF647 dye visualized with UV light. * shows the position of FTN2 variants and ** position of the residual dye.

To design the different FTN2_Azk_ variants, selection of theoretically suitable positions for Azk incorporation was carried out based on the following rules: The position must (1) be located within the first two-thirds of the protein from its N terminus (based on our observations for other model proteins), (2) be in a secondary structure-free region to avoid structural instability, (3) have A or G at +4 position [16], and (4) be surface and solvent accessible. Considering that FTN2 has two chains, buried positions upon heterodimerization of the two chains were excluded as well. In addition, one position within a region having secondary structures was also selected as negative control. The structure-free regions for FTN2 were determined based on the crystal structure of an anti-TNFα Fab (PDB code 5WUV). Surface available positions were determined using PDBePISA database [17]. The percentage of solvent accessibility for each position, of the original amino acid was calculated using CUPSAT database [18,19]. Based on these criteria, 11 positions were considered to be suitable, and all were selected for Azk incorporation into FTN2 (Table 1, Fig. 1A). The original codons of the selected positions were replaced by the amber codon (TAG) and then the resulting cassettes were inserted into the plasmid (pT7x3) (Fig. S1). The expression cassette of wt FTN2 is presented in Fig. 1B.

**Table 1:** Identified suitable positions for amber codon incorporation into FTN2 gene based on the defined rules. Nucleic acid (NA) for position +4 is in uppercase and bold. These data are obtained based on the X ray Chrystal structure of anti-TNFα Fab (PDB code 5WUV). Solvent accessibility for each position is calculated using CUPSAT and is valid for corresponding native amino acid and might be different upon substitution.

Position	Nucleic acid at position +4	AA context (position in structure free region)	Solvent accessibility for native AA [%]
**S**_**HC**_**133**	tag**A**ag	APS**Z**KSTSGC (4/10)	103.2
**K**_**HC**_**134**	tag**A**gc	APSS**Z**STSGC (5/10)	82.6
**E**_**HC**_**89**^a^	tag**G**at	RA**Z**DT	88.1
**G**_**HC**_**15**	tag**G**gc	QP**Z**GS (3/5)	57.2
**P**_**HC**_**41**	tag**G**gt	A**Z**GKG (2/5)	64.8
**N**_**LC**_**28**	tag**G**tt	SQ**Z**VGTN (3/7)	76.5
**G**_**LC**_**41**	tag**A**ag	KP**Z**KAP (3/6)	93.5
**S**_**LC**_**56**	tag**G**gt	Y**Z**GVPYR (2/7)	98.9
**Q**_**LC**_**100**	tag**G**gc	G**Z**G (2/3)	82.9
**T**_**LC**_**109**	tag**G**tt	R**Z**VAAP (2/6)	72.8
**E**_**LC**_**143**	tag**G**cg	YPR**Z**A (4/5)	93.9

a this AA sequence builds a helix and Z is incorporated in the middle of the helix structure.

### 2.2 Efficient production of wt FTN2 and FTN2_Azk_ variants in micro-bioreactor cultivations

A fed-batch like μ-cultivation procedure was used to investigate the cell growth kinetics and the product formation of the different plasmid-based FTN2 and FTN2_Azk_ production clones and to identify those amber codon positions best suited for Azk incorporation for FTN2_Azk_ production.

Regarding cell growth, BL21(DE3)(pT7x3_oFTN2) producing wt FTN2 grew to about ∼4 g/L cell dry mass (CDM) at the end of fermentation under non-induced condition (Fig. 1C). For all induced BL21(DE3)(pT7x3_oFTN2/oFTN2_Azk_) clones, scattered light signals used for CDM determination were slightly increased. The comparison between the clones with and without Azk treatment during the first 12 h of cultivation strongly indicates that the presence of 10 mM Azk does not adversely affect cell growth. BL21(DE3) without any plasmid reached a CDM of ∼7 g/L under same cultivation conditions (data not shown).

For all variants the specific FTN2 titers were measured, and the production levels obtained were comparable to that of wt FTN2 (Fig. S2). Possible misincorporation at the amber codon site was ruled out, as further explained in discussion, since no FTN2_Azk_ was produced in the negative control experiments with medium not supplemented with Azk exemplified by E_LC_143Z(−) (Fig. 1D). In addition, we also investigated a variant that has two Azk incorporation sites, the so-called double FTN2_Azk_ variant (S_LC_56Z-E_LC_143Z). We showed that it is feasible to incorporate two Azks on the same chain and maintain relatively high titer (3 mg/g CDM, Fig. S2).

### 2.3 Analytical characterization confirmed structure and functionality of FTN2_Azk_ variants comparable to wt FTN2

As there were no significant differences in product yields and cell growth among all our variants, we chose two variants for more detailed studies. Accordingly, we investigated the effect of Azk incorporation on the activity and structural perturbation in two variants, E_HC_89Z and E_LC_143Z, compared to wt FTN2. Azk was specifically introduced into a secondary structure region near the Complementarity Determining Region (CDR) in E_HC_89Z, while in E_LC_143Z, Azk was positioned in a structure-less region located further away from the CDR. The following important characteristic parameters were analyzed: surface accessibility, correct incorporation site, antigen-binding functionality, thermal stability, and possible structural perturbation. For that purpose, the Fabs were purified by affinity protein G chromatography. With protein G-based purification, the correctly folded Fabs, heterodimer form of HC and LC, were captured and the LC and LC homodimers (2LC) were efficiently excluded [20].

### 2.4 Selected Azk incorporation sites are surface accessible

**Fig. 2:**
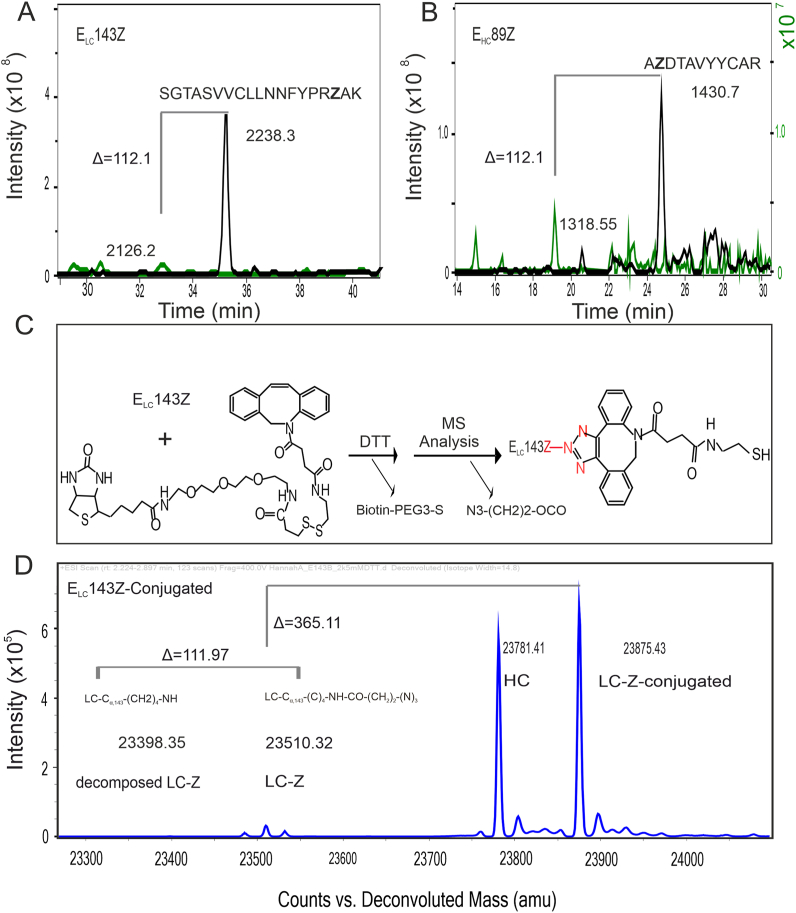
MS data on surface accessibility and site specific Azk incorporation of FTN2_Azk_ variants. **(A)** LC-ESI-MS/MS data of E_LC_143Z digested with endoproteinase, target fragment SGTASVVCLLNNFYPR**Z**AK (black line) and fragment with “potential MS borne” artefact (green line). **(B)** LC-ESI-MS/MS data of E_HC_89Z digested with trypsin, target fragment A**Z**DTAVYYCAR (black line) and fragment with “potential MS borne” artefact (green line). **(C)** Schematic representation of Azk decomposition during MS analysis (after DTT treatment): conjugation of LC through its Azk with DBCO-S-S-PEG3-Biotin. **(D)** Intact protein mass spectrometry analyses of E_LC_143Z variant conjugated with DBCO-S-S-PEG3-Biotin (see also Fig. S3 Aand B).

To determine the accessibility of Azk for click chemistry reactions, we attempted to conjugate the selected FTN2_Azk_ variants with DBCO-AF647 dye and DBCO-S-S-PEG3-Biotin (Disulfide biotin DBCO). In a first approach DBCO-AF647 dye was used for conjugation and visualization via gel electrophoresis. The results shown in Fig. 1E confirm Azk accessibility for click reaction and functionalization. Furthermore, adduct (conjugation with DBCO-S-S-PEG3-Biotin) formation and its abundance before and after DTT treatment was determined by MS analysis (Fig. S3 and Fig. 2C, D). According to our results (Fig. 2D), a sharp peak for conjugated LC-Z with a mass of 23875.4 Da was observed in the intact protein mode of mass analysis which resembles the expected mass of 23877.1 Da. A minor peak with a mass of 23510.3 Da was observed which can be assigned to non-conjugated LC-Z with intact Azk with an expected mass of 23510.1 Da. The results shown in Fig. S3 confirm high conjugation efficiency as almost only conjugated FTN2_Azk_ was detectable. Accordingly, all these data confirm the surface availability of the Azk for conjugation.

### 2.5 Site-specific Azk incorporation

Mass spectrometry was used to verify the site-specific incorporation of Azk into FTN2. In the course of these experiments, instability of Azk during mass spectrometric analysis was observed. In principle, replacing any AA with Azk leads to an increase in molecular weight (MW). For both studied variants, glutamic acid (E, MW = 147.13 Da) was substituted by Azk (Z, MW = 259.26 Da) resulting in an obvious signal peak shift in the mass of Δ = 112.13 Da. Presented in Fig. 2A and B the correct sizes of the FTN2_Azk_ variants (E_HC_89Z and E_LC_143Z) were found confirming the incorporation of Z at the expected position. In addition, for both variants (E_HC_89Z and E_LC_143Z) we identified a peak indicating that small fraction of the population having the same mass as wt FTN2 respectively. Besides this preferential E misincorporation, there was no indication for tyrosine (or other amino acids) misincorporation. The preferential E misincorporation is due to Azk decomposition during MS analysis, which is explained in details in Discussion section.

### 2.6 Antigen binding characteristics of FTN2_AzK_ variants

**Fig. 3:**
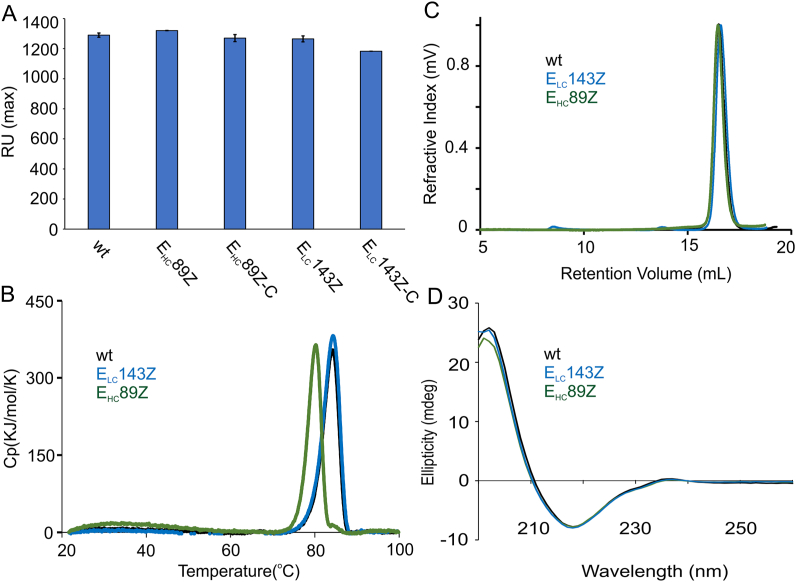
Characterization of functionality and structure of E_LC_143Z and E_HC_89Z variants compared to wt FTN2**. (A)** Antigen binding affinities of conjugated and non-conjugated variants via SPR analysis. (**B)** Heat capacity function versus temperature measured by DSC analyses. **(C)** Aggregation tendency measured by SEC-RALS analysis. Data were normalized against the concentration. (**D)** Secondary structure content evaluated by far CD analyses. Buffer background was subtracted from the raw spectra.

We used surface plasmon resonance (SPR) to characterize the antigen binding properties of the FTN2_Azk_ variants to the TNFα antigen, while wt FTN2 served as reference. According to our results, both FTN2_Azk_ variants in their non-conjugated and conjugated forms interacted with the immobilized antigen with the same strength (max. RU) as wt FTN2 (Fig. 3A, Fig. S4A). Homogeneities of the conjugated FTN2_Azk_ variants were confirmed by mass spectrometry analyses (Fig. S3).

In general, FTN2 interaction with its antigen is strong and stable. Based on our observations, no dissociation phases were observed for both the wt and FTN2_Azk_ variants (Fig. S4A). Reducing the amount of immobilized TNFα did not lead to a dissociation phase for non-conjugated wt FTN2 (Fig. S4B). Moreover, no dissociation was observed for the adducts as well, implying that the binding affinity of the variants in conjugated form remained the same as that of the wt FTN2 (Figs. S4C and D).

#### 2.6.1 Thermal stability, aggregation tendency and secondary structure content of FTN2_AzK_ variants

To test the influence of Azk incorporation on thermal stability of FTN2_Azk_ variants, we performed differential scanning calorimetry (DSC) studies. As the thermal denaturation is irreversible, the kinetic parameters were extracted by kinetic deconvolution of a single DSC scan using a non-linear least square-based algorithm described by Schön *et al.* [21]. According to the results, T_m_ for wt FTN2 and E_LC_143Z was the same (84.3 and 84.2, respectively) (Fig. 3B, Table 2). A slight decrease in ΔH and T_m_ was observed for E_HC_89Z. Other parameters like Δ*E*_a,_ and *k*_Tm_, independent from the amount of protein (temperature-dependent parameters), were almost the same.

**Table 2:** Thermodynamic parameters calculated for the wt FTN2 and FTN2_Azk_ variants based on DSC measurements. For wt FTN2 and E_LC_143Z, data were calculated from 3 or 2 DSC replicates. T_m_ (midpoint transient temperature), ΔH (unfolding enthalpy), ΔE_a_ (activation energy), (*K*_Tm_ denaturation rate). Purified wt FTN2 and FTN2_Azk_ variants were used for DSC analyses.

Variants	Tm (°C)	ΔH (Kcal/mol)	ΔEa (Kcal/mol)	K_Tm_ (min^−1^)
Wild type FTN2	84.33 ± 0.09	402.6 ± 2.4	141.5 ± 2.6	0.56 ± 0.01
E_LC_143Z	84.2 ± 0.23	389.35 ± 0.99	138.1 ± 0.7	0.54 ± 0.00
E_HC_89Z	80.3	342.6	170.6	0.69

According to SEC/RALS data, less than 3 % of the E_LC_143Z molecules were in non-native form after three months incubation at 4 °C in PBS. Whereas for wt FTN2 and E_HC_89Z, 100 % of the molecules were in a native form (Fig. 3C, Figs. S5A–C). Based on this observation, it could be concluded that for the selected positions, incorporation of Azk does not affect the denaturation rate or aggregation tendency. However, it should be mentioned that, with same setting, incorporation of 2 Azks in LC leads to higher tendency for higher molecular weight (HMW) species formation (2 Azks in LC, Fig. S5D).

As E_HC_89Z had similar antigen binding strength and slightly lower T_m_ and ΔH, we decided to monitor the possible structural changes for this variant with circular dichroism (CD) spectroscopy. Therefore, the secondary structure contents of the different variants were analyzed in the far-UV region. Accordingly, the far-UV spectra of FTN2 and FTN2_Azk_ variants exhibited one negative minimum at 208 nm and one positive maximum at 292 nm, which is characteristic for a β-sheet structured Fab. The overlay of the CD spectra indicates that the incorporation of Azk induces no significant structural changes in the secondary structure of FTN2_Azk_ variants (Fig. 3D). In addition, the structural content of the E_LC_143Z remained the same after 8 months incubation in PBS at 4 °C (Fig. S5E). This observation along with DSC data, confirms that the studied Fab can be chemically diversified via incorporation of Azk at the chosen positions.

## 3 Discussion

The incorporation of ncAAs into proteins at specific sites through amber stop codon suppression has become a common practice [22]. This concept is of particular interest for pharmaceutically active proteins such as Fabs, as it broadens the scope of these biopharmaceutical products and provides access to basic *in vivo* studies in the course of their production and application.

This study aimed to design Fab_Azk_ variants for efficient production in the periplasm of *E. coli,* while maintaining their functional and stable form. We utilized an amber suppressor tRNA_CUA_ to specifically recognize the amber codon, along with an aminoacyl-tRNA synthetase that facilitates the loading of Azk onto the cognate tRNA_CUA_^pyl^. We investigated the impact of Azk incorporated at different sites within an industry-relevant Fab, FTN2, as a model protein, on cell growth and expression levels. The goal was to assess the feasibility of producing fully functional and stable FTN2_Azk_ variants in *E. coli.*

In μ-bioreactor cultivations, the non-induced expression system BL21(DE3) (pT7x3_oFTN2) reached about 4 g/L biomass (Fig. 1C), which is 43 % less than for BL21(DE3). However, this is an expected observation, as (pT7x3_oFTN2) is a relatively large plasmid encoding for the orthogonal elements *Mm*PyIRS and *Mm*tRNA_CUA_^pyl^ thereby putting additional burden on cell metabolism. The observed increase in scattered light signal after induction does not indicate increased CDM as this effect can be assigned to changes in cell properties triggered by IB formation as reported previously [23]. Furthermore, results confirmed that the Azk concentrations applied in this study do not show any negative impact on cell growth (Fig. 1C).

Regarding specific titers, FTN2 showed an exceptional tolerance for Azk incorporation at several positions at both of its chains (Fig. 1A). All FTN2_Azk_ variants were successfully produced and no significant differences in product yields were observed compared to wt FTN2 (Fig. 1D, Fig. S2). These results are unexpected as production of ncAA modified proteins usually leads to lower expression levels compared to wt proteins [24]. A possible explanation for that observation could be the fact that Fabs belong to the group of challenging, difficult-to-produce proteins with in general low expression levels. For the double FTN2_Azk_ variant (S_LC_56Z-E_LC_143Z), it was shown that it is possible to incorporate two Azks into one LC (Fig. S2), an important observation that increases the potential of functionalization. However, it must be mentioned here that only about 60 % of the specific titer of variants with only one Azk, (S_LC_56Z and E_LC_143Z), could be achieved with this double FTN2_Azk_ variant. The ELISA used for quantification which explicitly detects only correctly folded Fabs, confirms the correct folding and structure of all FTN2_Azk_ variants. The negative control experiment with a cultivation medium not supplemented with Azk excludes potential misincorporation as no FTN2 was detectable via ELISA (Fig. 1D, Fig. S2).

In general, good expression yields gave us the opportunity to freely select FTN2_Azk_ variants with special features for more detailed studies on how Azk incorporation influences activity and structural content of FTN2. Thus, we selected a variant with Azk introduced to the LC (E_LC_143Z) and one with Azk in the HC (in E_HC_89Z). In addition, there is further variation as in E_LC_143Z, Azk is incorporated in a position far from CDR whereas in E_HC_89Z Azk is incorporated in a secondary structure region near to CDR.

The surface accessibility of Azk for functionalization is a key requirement for potential applications. Successful and efficient conjugation of Azk to DBCO-AF647 dye and DBCO-S-S-PEG3-Biotin was confirmed by gel electrophoresis (Fig. 1E) and MS analysis (Fig. 2 D and Fig. S3). These results confirm that the selection strategy that we implemented for identification of incorporation sites leads to FTN2_Azk_ variants with surface-accessible ncAA for click chemistry reactions. The observed conjugation efficiency in intact protein MS analysis (Fig. 2D and Fig. S3) was close to 100 %.

According to previous work, the misincorporation rate with our evolved orthogonal pair in presence of Azk is zero for glutamic acid (E) and tyrosine (Y) and, in absence of Azk the misincorporation rate is 33 % for tyrosine (supplementary information of [25]). Based on our observations (Fig. 2 A), at first glance, there is 6.2 % E misincorporation in E_LC_143Z. However, ELISA results from experiments without any Azk supplementation showed no FTN production (Fig. 1 D, Fig. S2) and thus misincorporation for Glutamic acid can be generally ruled out. We conclude that the peak with the mass of the wt FTN2 is an MS borne artefact. There are reports on instability of pAZF and other azide containing chemicals. According to literature [[26], [27], [28]]. N_2_ release from azide group leads to a peak shift in MS spectrum. In these reports, N_2_ release was either because of condition of the MS analyses (for -N3) or happened spontaneously under physiological conditions (for -pAZF). According to our observation, the mass shift of ∼112 Da cannot be due to N_2_ release. Considering the structure of Azk, decomposition at carbamate site would lead to a mass difference of 112 Da which exactly represents the observed difference (Fig. 2C and D). This conclusion is also supported by the results from intact protein MS analysis of DBCO-S-S-PEG3-biotin conjugated FTN2_Azk_ shown in Fig. 2D. In case of significant levels of E misincorporation a peak at mass of 23398.35 Da should be present. There is also no decomposition of Azk which can be explained that due to conjugation with DBCO-S-S-PEG3-biotin the carbamate site is possibly less exposed. Overall, the presence of the observed peaks could most likely be due to Azk decomposition. However, due to technical limitations, it is not possible to completely rule out the misincorporation.

Regarding the impact of Azk incorporation on antigen binding affinity, Islam *et al.* [1]. described negative effects when incorporation sites were located both inside and outside the CDR. The same observation was reported by Lindstedt *et al.* [29], namely that during antibody development, the incorporation of ncAA slightly increased the *K*_D_. According to our results of the SPR measurements (Fig. 3A), neither incorporation of Azk into E_HC_89Z and E_LC_143Z nor conjugation to DBCO-S-S-PEG3-biotin affect the antigen binding affinity. The variants bind to their antigen with same strength as the wt FTN2. We reported the maximum response unit as the measure of interaction strength. That is because wt FTN2 and the FTN2_Azk_ variants do not dissociate from the antigen and as reported by Katsamba *et al.* [30], the *K*_D_ (dissociation rate constant) could be calculated only when the decrease in signal is more than 5 %. As no dissociation happened, based on our data, it is not possible to calculate *K*_D_ (=*K*_d_/*K*_a_, dissociation equilibrium constants). Therefore, the maximum response unit was presented as a measure of interaction strength. To be more accurate, SPR measurement with lower target level of antigen immobilization was also performed and afterward, the affinity behavior of the FTN2_Azk_ variants was monitored. With this setting, almost no dissociation (less than 5 %) was observed as well (Fig. S4B). Furthermore, based on other reports when FTN2 is immobilized on the surface of the chip instead of antigen, a very strong interaction with no to very week dissociation phase was observed [31]. Overall, it can be concluded that FTN2 could be functionalized by Azk at different positions and these modifications are well tolerated and do not abrogate the binding affinity.

For characterization of conformational stability and possible perturbation in secondary structure, we employed DSC, SEC-RALS and CD. The correlation between conformational stability and pharmaceutical stability makes it possible to use the DSC method, which tests the conformational stability of the protein at a range of temperatures, to develop and screen the most stable candidates [32]. Although the aggregation tendency at low temperatures can be different [33,34], screening the more stable candidate through thermal denaturation is still appropriate [32]. The temperature at which a protein begins to unfold, midpoint transition temperatures (T_m_), activation energy (E_a_), and denaturation rate (*k*_Tm_) are the variables considered useful for predicting the long-term stability of antibodies. Accordingly, it has been reported that antibodies having higher T_m_ and E_a_ most likely have higher stability. According to our observations, the studied variants have a high T_m_ and considering the calculated kinetic parameters (K_Tm_, E_a_), the same long-term stability is to be anticipated for the studied FTN2_AZK_ variants as for the wt FTN2 (Fig. 3B, Table 2). Therefore, it can be concluded that Azk incorporation into FTN2 at these positions does not significantly affect the thermal stability and the variants can be considered thermally stable. Due to limitations in providing the required amount of sample material for DSC analysis, it was not possible to perform DSC analyses for conjugated forms of variants.

It is well known that the substitution of an amino acid in a protein affects its inherent tendency to aggregate [35]. Especially, electrostatic interactions play major roles in the self-association of peptides to form all form of aggregates [36]. As in our particular case, it is important to monitor the possible effect of substituting a charged amino acid (E, glutamic acid) with Azk, which has no charge at physiologic pH, we performed SEC-RALS analyses. To our knowledge, there is no report on effects of ncAA incorporation on the aggregation propensity of a modified protein, although this information can be relevant for pharmaceutical applications. With SEC-RALS studies, we showed that the FTN2_Azk_ variants were in the native folded state even after three months incubation in PBS (Fig. 3C). This is in accordance with expectations based on the kinetic parameters derived from DSC analyses (Table 2). However, incorporation of 2 Azks leads to higher tendency for HMW species formation (Fig. S5D).

As the E_HC_89Z variant, in which Azk is incorporated in a secondary structure region near to CDR region, has a slightly lower T_m_ and ΔH with similar antigen binding activity, we investigated, whether this difference was caused by a possible alteration in the secondary structure content. Therefore, the structure of wt FTN2 and FTN2_Azk_ variants was analyzed with CD. Interestingly, the spectrum for this variant at the same concentration was exactly matching that of wt FTN2 (Fig. 3D). Therefore, the observed change in the T_M_ of E_HC_89Z might be due to other variations in those interactions that do not affect the structural content. Overall, all FTN2_Azk_ variants showed same characteristics as the wt FTN2, even after longtime incubation, confirming that FTN2 tolerates incorporation of Azk at different positions.

## 4 Conclusion

We clearly demonstrate the possibility of chemical functionalization of proteins by incorporating ncAAs at well-considered sites. The investigated Fab FTN2 variants showed extremely high tolerance to the incorporation of Azk at different positions without compromising their production yield, binding affinity and structural properties. Even the conjugated FTN2_Azk_ variants displayed as strong interaction with their antigen as the non-conjugated variants. These results open up a wide range of possibilities to study the fate of functionalized proteins in the cell under physiological conditions, such as the *in vivo* proteolysis of a recombinant protein.

## 5 Materials and methods

### 5.1 Construction of modified oFTN2 cassettes

Stop codon (TAG, amber codon) was chosen as a codon for Azk incorporation at pre-determined positions in FTN2. Accordingly, one TAG codon is introduced into either the light chain (LC) or the heavy chain (HC) of the FTN2 gene. FTN2_Azk_ cassettes with two instances of Azk were also constructed where the Azks were either incorporated both in one and in two chains. Wild type FTN2 cassette within pET30a plasmid was used as starting material to replace preselected codons by the amber codon. Briefly, each of the new cassettes was constructed by doing whole-plasmid PCR using Q5 high fidelity DNA polymerase. To substitute the selected codon with the TAG, sets of primers, with the forward primers having TAG overhangs at their 5’ ends were used. After ligation step, the resulting plasmids were transformed into NEB5α. Then with the second set of primers the restriction sites for *Nde*I and *Bgl*II for each cassette were introduced. After plasmid amplification, the cassettes were excised using the restriction sites and were then inserted into pT7x3 plasmids (Fig. S1) which express the required orthogonal elements (*Mm*PylRS/*Mm*tRNA_CUA_^Pyl^ pair) for amber codon suppression [25]. Codon substitution for each cassette was confirmed by sequencing (Microsynth, Vienna, Austria). The competent BL21(DE3) cells were transformed with the plasmids and used for expression of FTN2_Azk_ variants. Afterward, master cell banks were prepared from the positive clones according to Ref. [37]. Details of all used materials are presented in Supplementary Table S1.

All plasmids with individual position of TAG codon were introduced by naming the substitution position (e. g. pT7x3_oFTN2. E_LC_143_am_: the plasmid expresses an oFTN2 variant that has amber codon (am) in its LC at position 143 at protein sequence level). All Azk incorporated variants (FTN2_Azk_) were introduced by naming the substitution position throughout this paper (e.g E_LC_143Z: a FTN2 variant with E to Z substitution at position 143 in light chain of FTN2). Details for all studied variants are presented in Supplementary Table S2.

### 5.2 Fed-batch-like cultivation in μ-bioreactor

The BioLector system (m2p-labs GmbH, Germany) was used for feed-batch-like μ-cultivations experiments in flower plates. The μ-cultivations were performed to verify the producibility of the FTN2_Azk_ variants and to evaluate the influence of different Azk incorporation positions on FTN2 production yield. Feed-in-time medium with enzymatic glucose release feature used for the cultivations [37]. All cultivations were performed at a shaking speed of 1400 rpm, at 30 °C and the relative humidity was kept at 80 %. The biomass was monitored online in 20 min intervals by scattered light measurement at 620 nm, and CDM was calculated from a pre-defined calibration curve. For inoculation, a defined volume of cell broth from the pre-culture [38] was transferred to achieve a final volume of 800 μL with an initial OD_600_ of 0.3. Induction was performed with a concentration of 0.5 mM of IPTG (Carl Roth, Germany) 12 h post-inoculation. The production phase was set to be 8 h, and endpoint samples were used for analyses.

### 5.3 Shake flask cultivation

As for the characterization of FTN2_Azk_ variants, a considerable amount of each variant is required, shake flask cultivations as well were carried out. To have fed-batch-like condition for shake flask-based experiments, the same medium composition as that used for the μ-cultivation, was used. Accordingly, cultivation was done at 30 °C with shaking speed of 200 rpm, induction with 0.5 mM of IPTG 12 h post-inoculation (100 mL medium in 500 mL baffled flasks). Production phase lasted 8 h and the biomass was collected for FTN2 variants extraction and purification.

### 5.4 Cell extraction

To confirm the production of the FTN2_Azk_ variants, biomass samples from μ-cultivation were lysed in 400 μl of lysis buffer (Tris-HCl 30 mM pH 8.2 containing 25 mM EDTA, 250 mM MgCl_2_, lysozyme (50 μl, 0.5 mg/ml), Benzonase (50 μl, 50 U/ml) and Triton-100X) according to Ref. [23]. After centrifugation at 15000 rpm at 4 °C for 15 min, the supernatant was collected to be used for ELISA analyses.

For samples from shake flask cultivations, after centrifugation step, the biomass was resuspended in the extraction buffer (100 mM Tris/HCl, 10 mM EDTA, pH 7.4). After complete resuspension, the samples were incubated at 60 °C for 1 h with shaking speed of 600 rpm. After centrifugation at 15000 rpm, 4 °C for 15 min, the supernatant was collected and filtered with a 0.22 μm filter and used for the purification process.

### 5.5 FTN2 ELISA

For sandwich ELISA briefly, anti-human IgG (Fab specific) goat antibody (I5260, 1:400, Sigma-Aldrich) was coated on the surface of the well (Nunc MaxiSorp, Roskilde, Denmark). Anti-human IgG mouse antibody (SA19255, 1:1000, sigma) and peroxidase-labeled anti-mouse IgG (Fab specific) goat antibody (A2304; 1:1000, Sigma-Aldrich) were used as first and secondary antibodies respectively. Details are provided by Ref. [23]. Specific titer was calculated based on the calculated CDM.

### 5.6 Purification

Protein G-based affinity chromatography (ÄKTA™ pure workstation, Cytiva, Sweden) in combination with a KanCap™ G (Kaneka, Japan) prepacked column was used for the purification of FTN2 variants according to Ref. [20]. Briefly, basic buffer (25 mM citrate, 25 mM Na_2_HPO_4_, pH 7.4) was used for capturing the FTN2 variants. Cation exchange buffer (25 mM citrate, 25 mM NaH_2_PO_4_, pH 2.3) was used to release the captured FTN2. The eluted FTN2 variants were immediately rebuffered using the PD-10 column (cytiva) and in case it was necessary, the volume was reduced with centrifugal ultrafilter (PES, 3KD, Sartorius) to have a concentrated sample. All variants were kept in PBS 0.01 M at 4 °C until use.

### 5.7 Site specific conjugation

Strain promoted azide alkyne cycloaddition (spAAc) chemistry (click chemistry) was used for site-specific conjugation of azide group of Azk with Disulfide Biotin DBCO or DBCO AF647 in PBS buffer at 25 °C with shaking speed of 600 rpm. Disulfide Biotin DBCO or DBCO AF647 were added to the purified FTN2_Azk_ variants at 4:1 ratio. The conjugated variants were used for monitoring the antigen binding affinity or visualization [25] of incorporated Azk.

### 5.8 Antigen binding analysis

For investigating how antigen binding ability of the FTN2_Azk_ variants is affected upon the incorporation of Azk or conjugation, interactions of FTN2_Azk_ variants and wild type FTN2 with TNFα were monitored by surface plasmon resonance (SPR, Biacore T200, GE Healthcare) analyses.

For coating the surface of the CM5 chip (Biacore Series S sensor) with the antigen, NHS/EDC chemistry was applied. To have theoretically 500 target level, 28 μg of purified TNFα (provided by Ref. [39]) was diluted in 10 mM sodium acetate pH 4.5 and then used for coating the surface of the chip. Running buffer for the immobilization step composed of PBS and 0.005 % *Polysorbate 20* (Tween 20). For the measurement phase, running buffer was composed of PBS, 0.1 % BSA, and 0.005 % Tween 20. Regeneration process was done using 3.3 M MgCl_2_ solution containing 5 mM EDTA [40]. All variants (conjugated and non-conjugated) were diluted in running buffer and their interactions with immobilized TNFα were monitored. To have the same condition, wt FTN2 and non-conjugated FTN2_Azk_ variants were kept at 25 °C at 600 rpm overnight but without Disulfide DBCO Biotin supplementation. In all measurements, contact time, dissociation time, and flow rate were set at 525 s, 350 s, and 40 μl/min, respectively.

### 5.9 Mass spectroscopy

For mass spectrometry analyses, LC-ESI-MS system (LC: Agilent 1290 Infinity II UPLC) was used to confirm the site-specific incorporation of Azk into FTN2. To confirm site-specific Azk incorporation, the purified FTN2_Azk_ variants were analyzed by peptide mapping procedure. Briefly, the samples were S-alkylated with iodoacetamide and then digested with either trypsin (for E_HC_89Z) or endoproteinase Lys-C (for E_LC_143Z). As a control, wt FTN2 was always analyzed. The digested samples were loaded on a nanoEase C18 column (nanoEase M/Z HSS T3 Column, 100 Å, 1.8 μm, 300 μm × 150 mm, Waters) using 0.1 % formic acid as the aqueous solvent. A gradient from 3.5 % B (B: 80 % ACN, 20 % A) to 40 % B in 30 min was applied, followed by a 5 min gradient from 40 % B to 95 % B that facilitates elution of large peptides, at a flow rate of 6 μL/min. Detection was performed with an iontrap MS (amazon speed ETD, Bruker) equipped with the standard ESI source in positive ion, DDA mode (= switching to MSMS mode for eluting peaks).

The same procedure with intact protein mode of analysis was used to monitor the conjugation efficiencies for the conjugated variants. Conjugated variants were analyzed in reduced (with DTT) and non-reduced forms. After loading the sample, a gradient from 15 to 80 % acetonitrile in 0.1 % formic acid (using a Waters BioResolve column (2.1 × 5 mm)) at a flow rate of 400 μL/min was applied (15-min gradient time). Detection was performed with a Q-TOF instrument (Agilent Series 6560 LC-IMS-QTOFMS) equipped with the Jetstream ESI source in positive ion, MS mode (range: 100–3200 Da). Instrument calibration was performed using ESI calibration mixture (Agilent). Data were processed using MassHunter BioConfirm B.08.00 (Agilent) and the spectrum was deconvoluted by MaxEnt.

### 5.10 Differential scanning calorimetry

10 μM of purified FTN2_Azk_ variants were prepared in PBS (pan Biotech) and used for DSC (PEAQ-DSC, Malvern Panalytical) analyses. All the measurements were performed according to the following settings: temperature range: 20–100 °C with 60 °C/h scan rate. PBS buffer was used as blank. Data analysis was performed by Ref. [21] using software developed in their laboratory. The software implements mixed model equations. All samples were used for DSC analyses within a week after the extraction and purification steps. Wild type FTN2 were used as control.

### 5.11 Analytical size exclusion chromatography (SEC) coupled with right-angle light scattering

SEC-RALS analysis was conducted on an OMNISEC multi-detector GPC/SEC (Malvern Panalytical, Worcestershire, UK) equipped with a refractive index-, right angle light scattering (RALS), and UV/VIS diode array detector.

Protein G affinity purified FTN2 variants (FTN2_Azk_ and wt FTN2) were separated on a Superdex S200 increase 10/300 GL column (Cytiva) maintained at 25 °C, using PBS as an isocratic mobile phase at a flow rate of 0.5 mL/min. The autosampler chamber was maintained at 25 °C. The injection volume varied between 20 and 100 μL according to sample concentration. The detectors were maintained at 25 °C. Protein concentration was measured online by using the refractive index detector. A dn/dc of 0.185 was taken. The instrument was calibrated using commercially available BSA (2 mg/mL) (Thermo Scientific™ Pierce™).

### 5.12 Electronic circular dichroism (ECD) spectroscopy

ECD spectra were collected on a Chirascan spectrometer (Applied Photophysics, Leatherhead, U.K.). The instrument was flushed with a nitrogen flow of 5 L min^−1^ throughout all experiments, and the temperature was kept constant at 25 °C. The samples were analyzed in the far-UV region (190–260 nm) at a scan speed of 10 s nm^−1^ and at a bandwidth of 1 nm. Cuvettes had a path length of 1 mm and were loaded with a protein concentration of 0.4 mg/mL. All measurements were performed in 5 mM phosphate buffer, pH 7.0. Data were analyzed with the PRO-DATA viewer software from Applied Photophysics.

## Data availability statement

Data included in article/supp. material/referenced in article.

## CRediT authorship contribution statement

**Hana Hanaee-Ahvaz:** Writing – original draft, Visualization, Methodology, Investigation, Formal analysis, Conceptualization. **Monika Cserjan-Pushmann:** Writing – review & editing, Validation, Supervision, Project administration, Methodology, Conceptualization. **Florian Mayer:** Investigation. **Christopher Tauer:** Investigation. **Bernd Albrecht:** Writing – review & editing, Conceptualization. **Paul G. Furtmüller:** Formal analysis. **Birgit Wiltschi:** Writing – review & editing, Validation, Conceptualization. **Rainer Hahn:** Writing – review & editing, Supervision, Formal analysis. **Gerald Striedner:** Writing – review & editing, Validation, Supervision, Resources, Funding acquisition, Formal analysis, Conceptualization.

## Declaration of competing interest

The authors declare that they have no known competing financial interests or personal relationships that could have appeared to influence the work reported in this paper.
