# Supplementary material for: Antibody fragments functionalized with non-canonical amino acids preserving structure and functionality - A door opener for new biological and therapeutic applications
Source: Heliyon. 2023 Nov 20;9(12):e22463. doi: 10.1016/j.heliyon.2023.e22463 (PMC10686840; doi:10.1016/j.heliyon.2023.e22463)
Supplement: Multimedia component 1 [file mmc1.docx]

***Heliyon***

**Supplemental Information**

**Antibody fragments functionalized with non-canonical amino acids preserving structure and functionality - a door opener for new biological and therapeutic applications**

Hana Hanaee-Ahvaz, Monika Cserjan-Pushmann, Florian Mayer, Christopher Tauer, Bernd Albrecht, Paul G. Furtmüller, Birgit Wiltschi, Rainer Hahn, Gerald Striedner

**Supplementary Table S1:** **Details of all used materials**

| **Bacterial strains** | | **Details** | **Source** |
| --- | --- | --- | --- |
| NEB5α | | Genotype: fhuA2Δ(argF-lacZ)U169 phoA glnV44 Φ80Δ(lacZ)M15 gyrA96 recA1 relA1 endA1 thi-1 hsdR17, (C2987) | from NEB |
| BL21(DE3) | | Genotype: fhuA2 [lon] ompT gal (λ DE3) [dcm] ∆hsdS, (C2527) | from NEB |
|  | | | |
| **Plasmids** | |  |  |
| pET30a_oFTN2 | | Our Previous study | ^23^ |
| pT7x3 | General description | Ori: pET, expresses *Mm*PyIRS under the control of T7, *Mm*tRNA_CUA_^Pyl^ under the control of T7-proK | ^25^ |
|  | proKp-MmtRNA_CUA_^Pyl^ | AGGCATTTTGCTATTAAGGGATTGACGAGGGCGT  ATCTGCGCAGTAAGATGCGCCCCGCATTGGAAACCTGATCATGTAGATCGAATGGA  CTCTAAATCCGTTCAGCCGGGTTAGATTCCCGGGGTTTCCGCCAAATTCGAAAAGCC  TGCTCAACGAGCAGGCTTTTT | DNA sequence |
|  | MmPylRS | MDKKPLNTLISATGLWMSRTGTIHKIKHHEVSRSKIYIEMACGDHLVV  NNSRSSRTARALRHHKYRKTCKRCRVSDEDLNKFLTKANEDQ  TSVKVKVVSAPTRTKKAMPKSVARAPKPLENTEAAQAQPSGSKFSPA  IPVSTQESVSVPASVSTSISSISTGATASALVKGNTNPITSMS  APVQASAPALTKSQTDRLEVLLNPKDEISLNSGKPFRELESELLSRRKK  DLQQIYAEERENYLGKLEREITRFFVDRGFLEIKSPILIPL  EYIERMGIDNDTELSKQIFRVDKNFCLRPMLAPNLYNYLRKLDRALPDP  IKIFEIGPCYRKESDGKEHLEEFTMLNFCQMGSGCTRENLE  SIITDFLNHLGIDFKIVGDSCMVYGDTLDVMHGDLELSSAVVGPIPLDR  EWGIDKPWIGAGFGLERLLKVKHDFKNIKRAARSESYYNGI  STNL | Protein sequence |
|  | | | |
| **Primers** | | **Sequence** |  |
| N28-LC-F  N28-LC-R | | TAGGTTGGCACCAATGTTGC  TTGGCTGGCTTTACACGTG | IDT |
| G41-LC-F  G41-LC-R | | TAGAAGGCTCCGAAAGCG  AGGCTTTTGCTGATACCAC | IDT |
| S56-LC-F  S56-LC-R | | TAGGGTGTGCCGTATCGCTT  ATACAGGAAGCTGGCGCT | IDT |
| Q100-LC-F  Q100-LC-R | | TAGGGCACGAAAGTTGAG  ACCAAAGGTCAGCGGATAAATA | IDT |
| T109-LC-F  T109-LC-R | | TAGGTTGCAGCGCCGAG  ACGCTTGATCTCAACTTTCGTG | IDT |
| S127-LC-F  S127-LC-R | | TAGGGTACGGCCAGCGTTG  CTTCAGCTGTTCGTCGCTA | IDT |
| E143-LC-F  E143-LC-R | | TAGGCGAAAGTCCAGTGGA  ACGTGGGTAAAAGTTATTCAGC | IDT |
| G15-HC-F  G15-HC-R | | TAGGGCAGCTTGCGTCTGTC  CGGCTGGACCAGGCC | IDT |
| P41-HC-F  P41-HC-R | | TAGGGTAAGGGTTTGGAGTGGAT  CGCCTGGCGCACCCA | IDT |
| E89-HC-F  E89-HC-R | | TAGGATACCGCAGTGTATTACTGC  TGCGCGCAGGCTGTT | IDT |
| S133-HC-F  S133-HC-R | | TAGAAGAGCACTAGCGGTGGC  GCTCGGCGCCAGCGG | IDT |
| K134-HC-F  K134-HC-R | | TAGAGCACTAGCGGTGGC  GCTGCTCGGCGCCAG | IDT |
| pT7x3_BglII_ amp | | CTAAAGATCTAATTCCTAGCATAACC | IDT |
| pT7x3_NdeI_ | | GAGACATATGTAATTCTCCTTCTTAAAGTTAAAC | IDT |
|  | | | |
| **Chemicals** | | | |
| N^6^-[(2-Azidoethoxy)carbonyl]-L-lysine | | Iris Biotech GmbH (Marktredwitz, Germany) | |
| Molecular biology chemicals (Restriction enzymes (NdeI and BglII, DpnI, Q5^®^ DNA polymerase, T4 DNA ligase, DNA and plasmid purification kits) | | New England Biolabs (NEB, Ipswich, USA) | |
| TNFα | | From ^37^ | |
| BSA | | Sigma-Aldrich (St. Louis, MO). |  |
| Polysorbate 20 (Tween 20) | | Sigma-Aldrich (St. Louis, MO). | |
| EDTA | | Sigma-Aldrich (St. Louis, MO). | |
| MgCL_2_ | | Sigma-Aldrich (St. Louis, MO). | |
| Na_2_HPO_4_ | | Sigma-Aldrich (St. Louis, MO). | |
| NaH_2_PO | | Sigma-Aldrich (St. Louis, MO). | |
| Tris/HCl | | Sigma-Aldrich (St. Louis, MO). | |
| lysozyme | | Sigma-Aldrich (St. Louis, MO). | |
| citrate | | Sigma-Aldrich (St. Louis, MO). | |
| Disulfide Biotin DBCO | | Jena Bioscience | |
| DBCO AF647 | | Jena Bioscience | |
| Benzonase | | Sigma-Aldrich (St. Louis, MO). | |
| Lysozyme | | Sigma-Aldrich (St. Louis, MO). | |
| anti-human IgG (Fab specific) goat antibody ( | | Sigma, I5260 | |
| Anti-human IgG mouse antibody ( | | Sigma, SA19255 | |
| anti-mouse IgG (Fab specific) goat antibody | | Sigma, A2304 | |
| KanCap^TM^ G column | | Kaneka, Japan | |

**Supplementary Table S2: FTN2Azk varians.**

| for all variants: o: ompA^ss^ signal sequence, am: amber codon, Z and Azk one and 3 letter code for N^6^-[(2-Azidoethoxy)carbonyl]-L-lysine and the plasmid expresses FTN2 without or with amber codon at mentioned positions | | |
| --- | --- | --- |
|  | **Description, Expressed variant** |  |
| pT7x3_oFTN2. | control / wt FTN2 | this study |
| pT7x3_oFTN2.G_HC_15_am_ | oFTN2 with G15 in Heavy chain (HC) substituted by Z, G_HC_15Z | this study |
| pT7x3_oFTN2.P_HC_41_am_ | oFTN2 with P41 in HC substituted by Z, P_HC_41Z | this study |
| pT7x3_oFTN2.E_HC_89_am_ | oFTN2 with E89 in HC substituted by Z, E_HC_189Z | this study |
| pT7x3_oFTN2.S_HC_133_am_ | oFTN2 with S133 in HC substituted by Z, S_HC_133Z | this study |
| pT7x3_oFTN2.K_HC_134_am_ | oFTN2 with K134 in HC substituted by Z, K_HC_134Z | this study |
| pT7x3_oFTN2. E_LC_143_am_ | oFTN2 with E143 in light chain (LC) substituted by Z, E_LC_143Z | this study |
| pT7x3_oFTN2. T_LC_109_am_ | oFTN2 with T109 in LC substituted by Z, T_LC_109Z | this study |
| pT7x3_oFTN2. Q_LC_100_am_ | oFTN2 with Q100 in LC substituted by Z, Q_LC_100Z | this study |
| pT7x3_oFTN2. S_LC_56_am_ | oFTN2 with S56 in LC substituted by Z, S_LC_56Z | this study |
| pT7x3_oFTN2. G_LC_41_am_ | oFTN2 with G41 in LC substituted by Z, G_LC_41Z | this study |
| pT7x3_oFTN2. N_LC_28_am_ | oFTN2 with N28 in LC substituted by Z, N_LC_28Z | this study |
| pT7x3_oFTN2.E_HC_89_am_-E_LC_143_am_ | Variants having 2 Azks, one in HC and one in LC | this study |
| pT7x3_oFTN2.S_HC_133_am_-E_LC_143_am_ | Variants having 2 Azks, one in HC and one in LC | this study |
| pT7x3_oFTN2.E_HC_89_am_-S_HC_133_am_ | Variants having 2 Azks, both in HC | this study |
| pT7x3_oFTN2.S_LC_56_am_-E_LC_143_am_ | Variants having 2 Azks, both in LC | this study |

**Supplementary Figure S1**

A


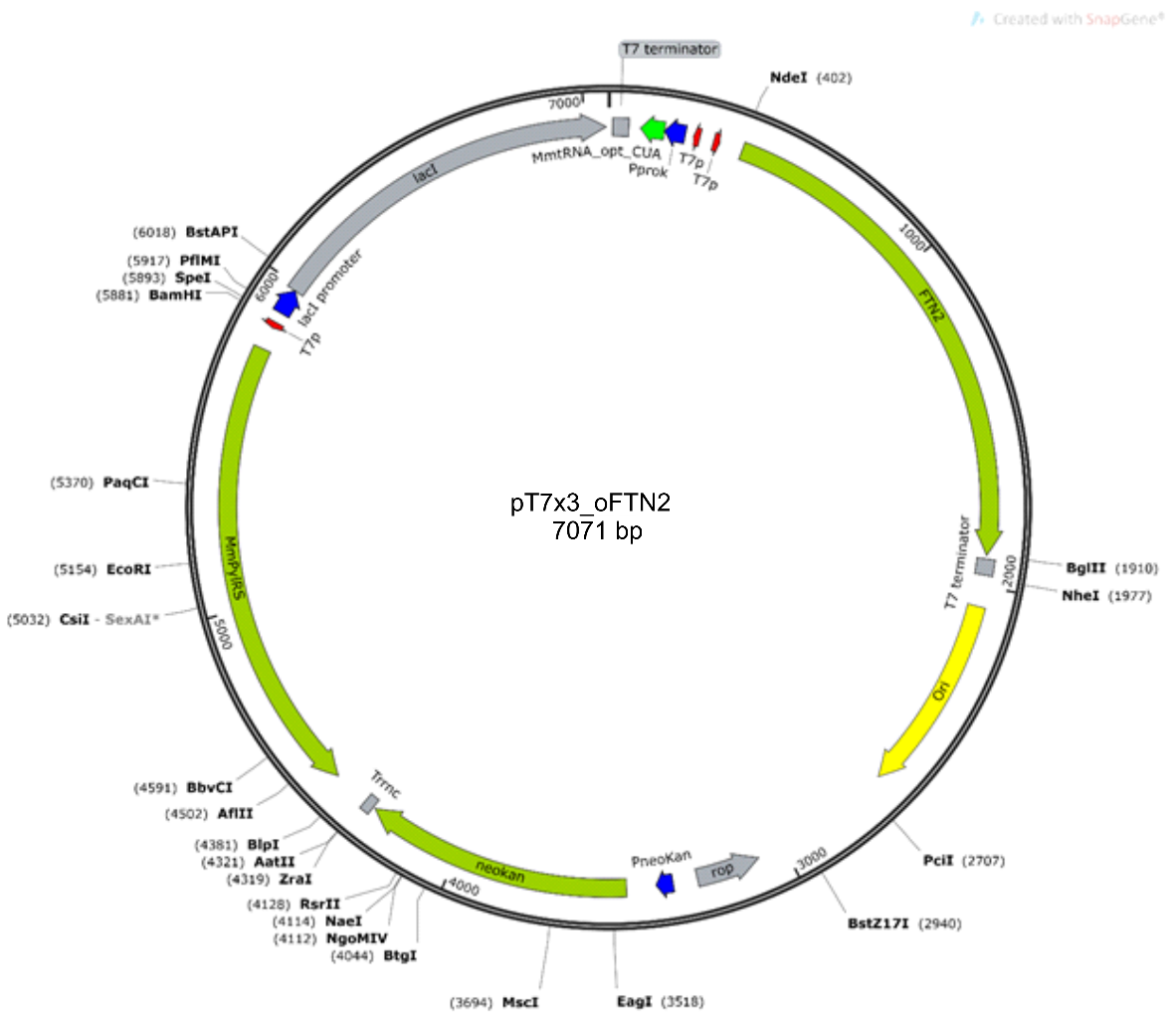


B


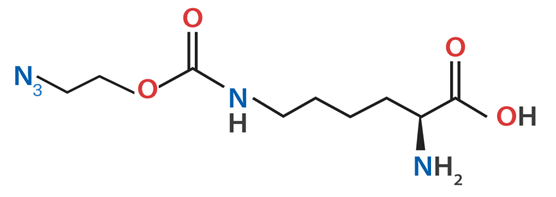


**Supplementary Figure S1.** Related to Figure 1. **A)** Map of pT7x3 plasmid expressing the required orthogonal elements (*Mm*PylRS and *Mm*tRNA_CUA_^Pyl^). The oFTN2 cassette with or without amber codon was inserted using NdeI and BglII restriction sites**.****B)** Chemical structure of Azk (N6-[(2-Azidoethoxy)carbonyl]-L-lysine).

**Supplementary Figure S2**


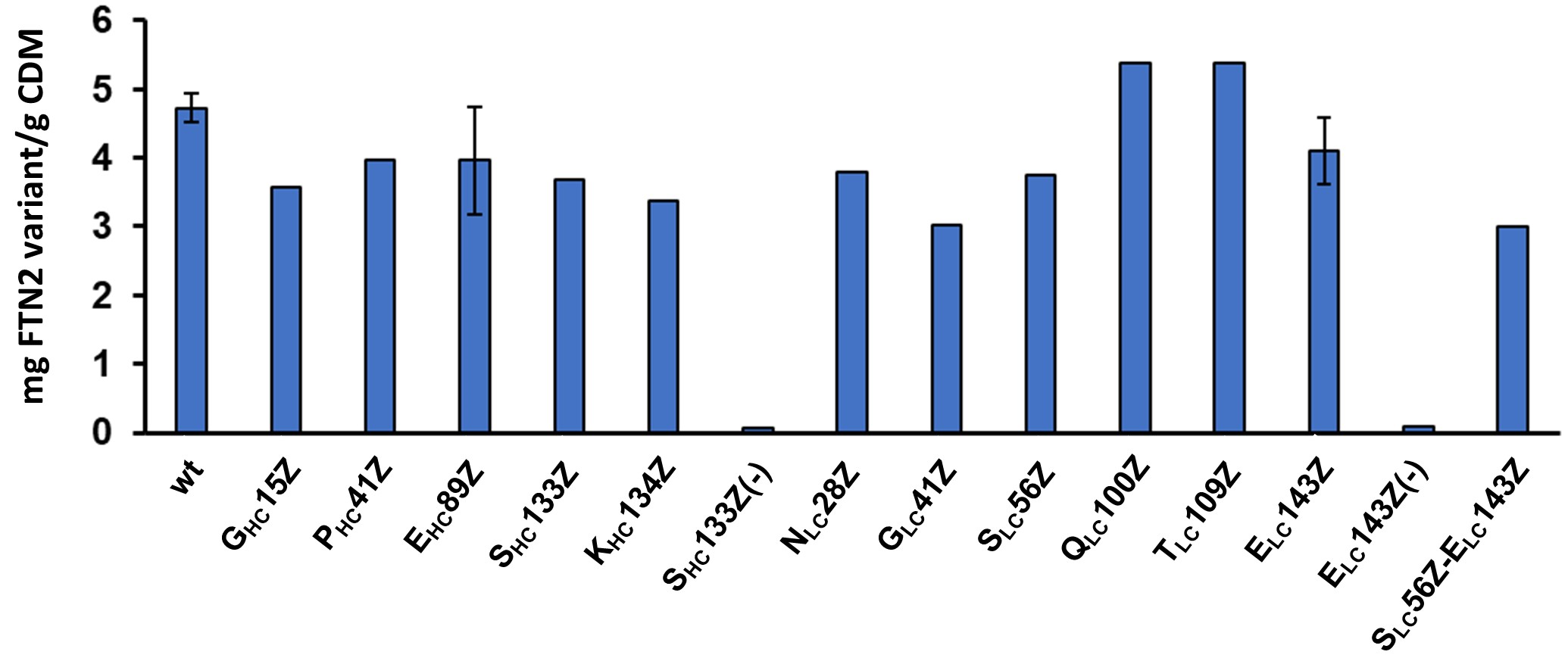


**Supplementary Figure S2.** Related to Figure 1D. Expression levels of FTN2_Azk_ variants grown in µ-scale fedbatch like cultures. For all cultures with single azk variants (except for S_HC_133Z(-) and EHC143Z(-) which served as reference) the medium was supplemented with 10 mM Azk and the culture medium for the double Azk variant (S_LC_56Z-E_LC_143Z) with 20 mM Azk. Induction was performed with 0.5 mM IPTG 12 hours after inoculation. Specific titers for E_HC_89Z, E_LC_143Z and wt were calculated using two biological replicates, while all others were generated using one biological replicate. Error bars were calculated from deviation from the mean.

**Supplementary Figure S3**

A.


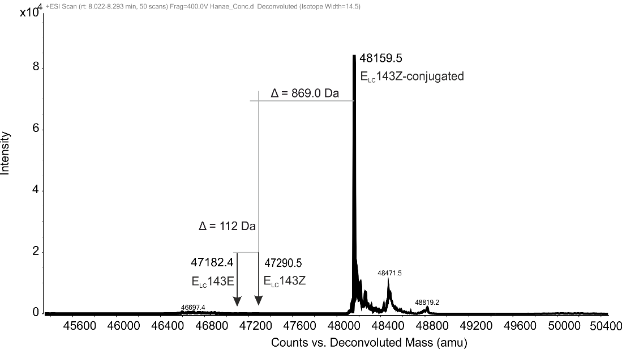


B.


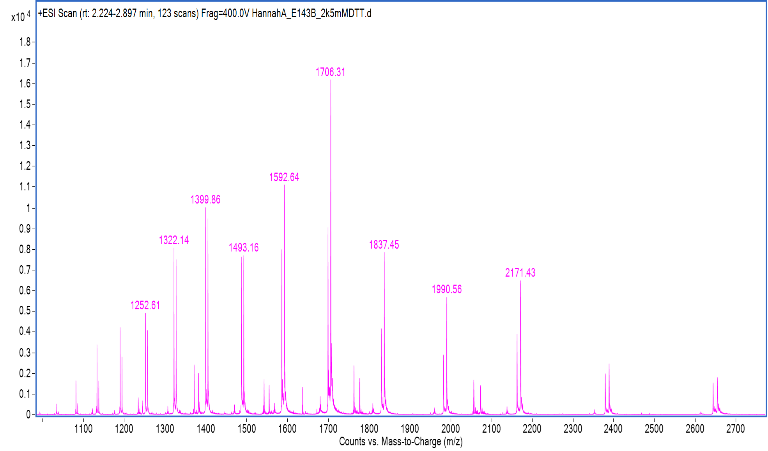


**Supplementary Figure S3.** Related to Figure 2. **A)** Mass spectrometry analyses. The conjugation efficiency with strain promoted azide alkyne cycloaddition chemistry was shown by mass spectrometry analyses with intact protein procedure. Conjugation leads to 869 Da shift (47290.5 Da for non-conjugated and 48159.5 Da for conjugated, position). For this experiment 4:1 ratio (DBCO-S-S-PEG3-Biotin: FTN2 variants) was applied and the reaction was performed overnight and was not treated with DTT. **B)** Primary spectrum with multiple charges that used for preparing deconvulted spectrum.

**Supplementary Figure S4**


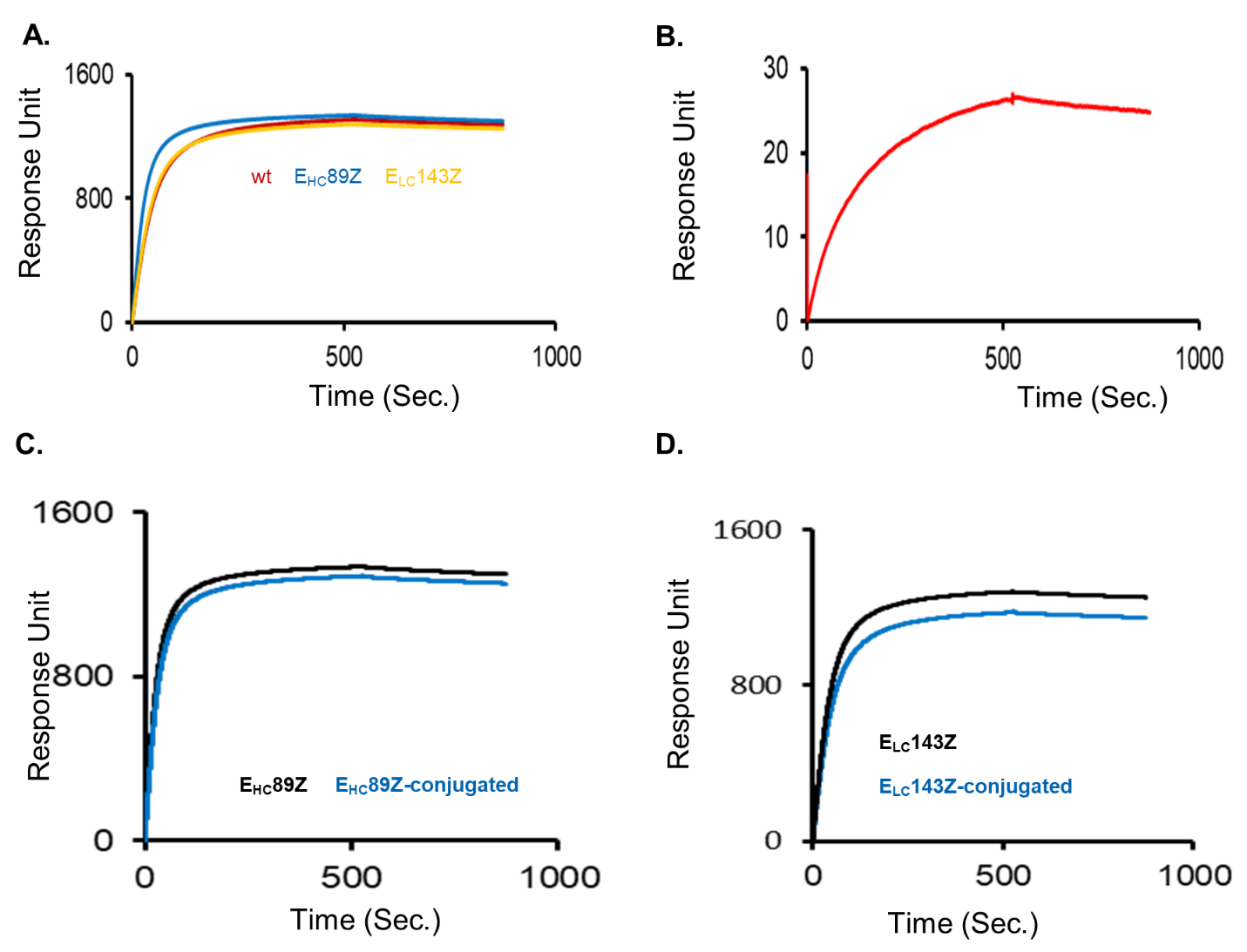


**Supplementary Figure S4.** Related to Figure 3A. Antigen binding affinity of FTN2 variants (conjugated and non-conjugated), TNFα is immobilized on the surface of the chips. A) All FTN2 variants show strong avidity behavior. Target level set for 500. 50 nM of FTN2 variants were used for the measurement. B) A very weak dissociation phase was observed for wt FTN2 when the target level set for 50. C) and D) Sensograms of the conjugated and non-conjugated forms of FTN2 variants. TNFα is immobilized for target level of 500. 50 nM of FTN2 variants were used for the measurement. Conjugation condition: FTN2 variant to linker ratio 1:4, overnight incubation at RT and shaking rate 800 rpm. The conjugated forms of the FNT2_Azk_ variants showed the same binding strength as the non-conjugated forms.

**Supplementary Figure S5**


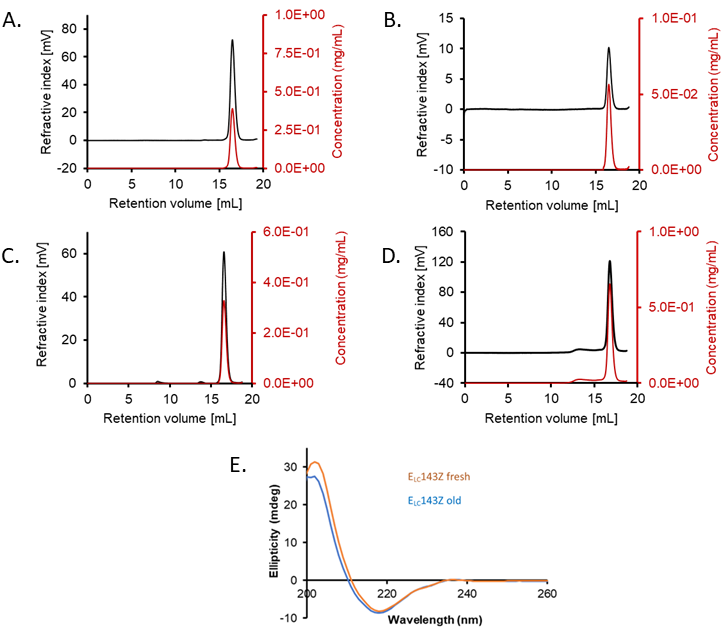


**Supplementary Figure S5.** Homogenity and structural stability of FTN2_Azk_ variants. SEC/RALS data of **(A)** wt FTN2 (15 µL, 1.65 mg/mL), **(B)** E_HC_89Z (10 µL, 0.9 mg/mL), **(C)** E_LC_143Z (90 µL, 0.25 mg/mL) and **(D)** S_LC_56Z-E_LC_143Z (45 µL, 0.76 mg/mL). Concentration presented with the second y axis. **(E)** Far UV CD analyses for monitoring the secondary structure content of FTN2 _Azk_ variants. In all experiment the variants were in PBS buffer (5 mM, pH 7.4) at 0.4 mg/mL and cuvette with 1 mm path length was used. Old sample were incubated for 8 months in PBS at 4°C.

References:

23. Fink, M., Vazulka, S., Egger, E., Jarmer, J., Grabherr, R., Cserjan-Puschmann, M., and Striedner, G. (2019). Microbioreactor Cultivations of Fab-Producing Escherichia coli Reveal Genome-Integrated Systems as Suitable for Prospective Studies on Direct Fab Expression Effects. Biotechnology Journal *14*, 1800637. <https://doi.org/10.1002/biot.201800637>.

25. Galindo Casas, M., Stargardt, P., Mairhofer, J., and Wiltschi, B. (2020). Decoupling Protein Production from Cell Growth Enhances the Site-Specific Incorporation of Noncanonical Amino Acids in E. coli. ACS Synthetic Biology *9*, 3052-3066. 10.1021/acssynbio.0c00298.

37. Lingg, N., Kröß, C., Engele, P., Öhlknecht, C., Köppl, C., Fischer, A., Lier, B., Loibl, J., Sprenger, B., Liu, J., et al. (2022). CASPON platform technology: Ultrafast circularly permuted caspase-2 cleaves tagged fusion proteins before all 20 natural amino acids at the N-terminus. New Biotechnology *71*, 37-46. <https://doi.org/10.1016/j.nbt.2022.07.002>.
